# ICG‐fluorescence angiography assessment of colon interposition for oesophageal cancer – A Video Vignette

**DOI:** 10.1111/codi.16076

**Published:** 2022-03-02

**Authors:** Hidde A. Galema, Jan M. van Rees, Robert A. Matthijsen, Denise E. Hilling, Bas P. L. Wijnhoven, Sjoerd M. Lagarde

**Affiliations:** ^1^ Department of Gastrointestinal and Oncological Surgery Erasmus MC Cancer Institute Rotterdam The Netherlands; ^2^ Department of Surgery Elisabeth – TweeSteden Hospital Tilburg The Netherlands

Oesophageal cancer surgery is complex and carries a high risk of complications. Nowadays, a gastric conduit is the reconstruction of choice after oesophageal resection. In a limited percentage of cases a gastric conduit is not possible. Previous gastric surgery, tumour location, or iatrogenic injury to the gastroepiploic artery can all be reasons to choose a different interposition. In these cases, a colonic interposition is the best alternative for continuity restoration. This Video S1 vignette demonstrates how indocyanine green (ICG) fluorescence angiography can be used to assess perfusion of the colon interposition.

The case reported here is of a 63‐year old woman who was diagnosed with oesophageal cancer for which she received neoadjuvant chemoradiotherapy followed by Ivor Lewis oesophagectomy with gastric conduit reconstruction. The procedure was complicated by a diaphragmatic hernia and an intrathoracic colon. During repair of this diaphragmatic hernia, the gastroepiploic artery was injured, leaving the gastric conduit without sufficient blood supply and necrosis as a consequence. The gastric conduit was resected, and a temporary oesophageal fistula was performed. The patient was referred to an academic medical centre for delayed restoration of continuity with a colon interposition. During surgery, 10 mg ICG was intravenously injected and an intense fluorescence signal over the whole left colon was observed. After transection of the colon, another 10 mg ICG was administered to assess perfusion of the colon interposition. Perfusion was judged as sufficient and adequate anastomoses were created. Postoperatively the patient recovered well and was able to take full oral intake after 7 weeks.

## CONFLICT OF INTEREST

All authors report no conflicts of interest.

## AUTHOR CONTRIBUTIONS

*Conceptualization*: Hidde A. Galema, Jan M. van Rees, Sjoerd M. Lagarde. *Software*: Hidde A. Galema. *Supervision*: Sjoerd M. Lagarde. *Writing – Original Draft Preparation*: Hidde A. Galema, Sjoerd M. Lagarde. *Writing – Review & Editing*: Jan M. van Rees, Robert A. Matthijsen, Denise E. Hilling, Bas P. L. Wijnhoven.

## Supporting information

Video S1

